# Neutrophils Promote Tumor Progression in Oral Squamous Cell Carcinoma by Regulating EMT and JAK2/STAT3 Signaling Through Chemerin

**DOI:** 10.3389/fonc.2022.812044

**Published:** 2022-01-28

**Authors:** Xiaoyuan Hu, Fenggang Xiang, Yuanyong Feng, Fei Gao, Shengyou Ge, Chengqin Wang, Xuan Zhang, Ning Wang

**Affiliations:** ^1^Department of Pathology, School of Basic Medicine, Medical College of Qingdao University, Qingdao, China; ^2^Department of Pathology, Pingxiang People’s Hospital, PingXiang, China; ^3^Department of Pathology, the Affiliated Hospital of Qingdao University, Qingdao, China; ^4^Department of Oral and Maxillofacial Surgery, School of Stomatology and The Affiliated Hospital of Qingdao University, Qingdao, China

**Keywords:** chemerin, oral squamous cell carcinoma, neutrophils, JAK2/STAT3, EMT, tumor progression

## Abstract

Oral squamous cell carcinoma (OSCC) is the most common malignancy of the oral cavity. In the tumor microenvironment, tumor-associated neutrophils (TANs) can promote tumor growth, invasion, and metastasis. The aim of our study was to explore the relationship between neutrophils infiltration and Chemerin expression in tumor cells, as well as their relationship with the clinicopathological parameters and clinical prognosis of 74 cases of OSCC. We also explored the role of the interaction between neutrophils and Chemerin in the functions of OSCC cells (Cal27, SCC9, and SCC15) *in vitro*. Our results showed that in OSCC, Chemerin over-expression may increase neutrophils infiltration in tumor tissues. Chemerin over-expression and neutrophils infiltration were the prognostic factors of poor clinical outcomes. Furthermore, we discovered that neutrophils promoted OSCC migration, invasion, and proliferation and EMT through Chemerin. Neutrophils activated JAK2/STAT3 signaling through Chemerin and then up-regulated its downstream signaling target genes, such as Phospho-Rb, E2F1, CyclinE1, and CyclinD1. Taken together, our results revealed that neutrophils and Chemerin are potentially involved in OSCC progression and metastasis. Neutrophils may promote the JAK2/STAT3 signaling pathway and EMT in OSCC cells through Chemerin.

## Introduction

Oral squamous cell carcinoma (OSCC) is the most common malignancy of the oral cavity (1). Although treatment methods, such as chemotherapy, radiotherapy, and surgical therapy, have advanced in recent years, the 5-year survival rate of patients with OSCC has remained less than 60% (2). The mechanisms of the occurrence and development of OSCC are still unclear. However, recent studies have shown that the interaction between tumor cells and immune cells creates a favorable microenvironment for cancer initiation, progression, and metastasis (3, 4). The cross-talk between tumor cells and immune cells may promote tumor development (4).

Recent studies have revealed that in addition to tumor cells, inflammation and the immune system, as indispensable participants in tumor formation, provide an attractive environment for tumor growth and metastasis. Neutrophils are not only the most abundant circulating white blood cells, they are also one of the main infiltrating immune and inflammatory cells in OSCC (5). A growing body of research has demonstrated that tumor-associated neutrophils (TANs), a predominant component of the tumor microenvironment, participate in tumor initiation, growth, proliferation, and metastatic spread (6). In accordance with different tumor microenvironments and in addition to the contact mechanism, TANs can induce the paracrine release of cytokines and chemokines with tumor-promoting or antitumor functions to affect tumor progression (7). Evidence showing that increased TANs infiltration is associated with poor clinical outcomes in OSCC exists (8, 9).These novel aspects of neutrophils biology may contribute to OSCC progression and metastasis. However, only a few studies have been done on the specific signaling pathways and molecular mechanisms involved in the interaction between neutrophils and OSCC (10).

EMT is a key event where in epithelial cells lose their epithelial characteristics and acquire a mesenchymal phenotype (11). Its phenotypic transition is manifested as the loss of cell polarity and cell–cell connection and the acquisition of mesenchymal characteristics, such as motility and invasiveness, by epithelial cells (12). At the molecular level, it usually manifests as the decreased expression of E-cadherin and the increased expression of N-cadherin, mesenchymal markers (vimentin), and transcription factors (Slug and Snail) (12–14). Recent studies have illustrated that EMT plays a vital role in tumor progression and metastasis (15–17). Stromal cells in the tumor microenvironment can induce EMT. Neutrophils constitute an important part of tumor stroma, and they are mainly involved in regulating tumor progression (6, 18). However, studies on neutrophils function in EMT and its possible molecular mechanisms remain rare.

Chemerin, an effective chemoattractant protein encoded by retinoic acid receptor responder 2, was first discovered in psoriatic skin lesions (19, 20). Recent studies have revealed that the expression of Chemerin is dysregulated in several types of tumors. Chemerin expression is up-regulated in neuroblastoma, esophageal squamous cell carcinoma, and OSCC (20–22) but is down-regulated in hepatocellular carcinoma and adrenocortical carcinoma (23, 24). Weigert et al. (25) reported that Chemerin levels in the serum of patients with inflammatory bowel disease are elevated relative to those in the serum of healthy controls, indicating that Chemerin has a potential regulatory function in intestinal inflammation. In addition, Sotiropoulos et al. (8) reported that Chemerin,a biomarker of nonsmall cell lung cancer, is involved in tumor-promoting networks and inflammatory and cancer-related metabolic pathways.

Our previous studies demonstrated that Chemerin is over-expressed in OSCC (26), that TANs infiltration in tumor tissues is increased (9), and that both of these factors are correlated with the poor clinical outcomes of patients with OSCC (9, 22). Chemerin expression in tumor cells may play important roles in immune surveillance. However, the role of Chemerin and neutrophils in OSCC remains unknown. The JAK2/STAT3 pathway has been found to play a critical role in the progression of a variety of tumors (27–29). The expression levels of STAT3 and phosphorylated STAT3 in OSCC are increased compared with those in normal tissues (30). A number of previous findings strongly suggest that the persistent activation of STAT3 in head and neck squamous cell carcinomas, accompanied by increases in STAT3 tyrosine phosphorylation, is linked to cell proliferation, differentiation, and apoptosis (31, 32). The role of neutrophils, along with JAK2/STAT3 signaling and EMT,in OSCC has been poorly addressed. Therefore, the purpose of our work is to determine how neutrophils activate the JAK2/STAT3 pathway in OSCC and how Chemerin and neutrophils promote the migration, invasion, and proliferation of OSCC.

In the present study, we studied the relationship between TANs infiltration and Chemerin expression. We also investigated TANs infiltration and Chemerin expression in relation to clinicopathological parameters and clinical prognosis. Moreover, we explored the roles of neutrophils and Chemerin in EMT and JAK2/STAT3 signaling pathway regulation and identified the downstream targets phosphor-Rb, E2F1, CyclinD1, and CyclinE1. Our results indicated that neutrophils could regulate the JAK2/STAT3 signaling pathway and EMT through Chemerin to promote OSCC and provided support that neutrophils and Chemerin are potential therapeutic targets for the treatment of OSCC.

## Material and Methods

### Patients and Specimens

A total of 74 patients (51 males and 23 females) with primary tongue squamous cell carcinoma treated at the Affiliated Hospital of Qingdao University between 2005 and 2010 participated in this study. The clinical pathological data of all the patients were complete, and no radiotherapy or chemotherapy was performed before surgery. The clinicopathological information of the patients is presented in **Table 1**. The study protocol was approved by the ethics committee of the Affiliated Hospital of Qingdao University. Written informed consent was obtained from all of the patients and healthy controls. The studies were conducted in accordance with the Declaration of Helsinki.

**Table 1:** The relationship between the expression of Chemerin, CD15 + TANs density and clinicopathological parameters in OSCC (x / %).

Variables		n	@ 1	@ 2	@ 3	*Z*	*P value*
**Sex**	Male					-0.44	0.965
	Female	51	11 (21.57)	20 (39.22)	20 (39.22)		
		23	6 (26.09)	7 (30.43)	10 (43.48)		
**Age**						-1.225	0.221
	≤60 years	40	8 (20.00)	13 (32.50)	19 (47.50)		
	>60 years	34	9 (26.47)	14 (41.18)	11 (32.35)		
**TNM stage**						-4.293	0.000*
	I,II	37	14 (37.84)	17 (45.95)	7 (18.92)		
	III,IV	37	2 (5.41)	12 (32.43)	23 (62.16)		
**Differentiation**						-0.685	0.493
	G1,G2	34	8 (23.53)	10 (29.41)	16 (47.06)		
	G3	40	9 (22.50)	17 (42.50)	14 (35.00)		
**Lymph node metastasis**						-4.789	0.000*
	Yes	34	1 (2.94)	10 (29.41)	23 (67.65)		
	No	40	16 (40.00)	17 (42.50)	7 (17.50)		
**Tumor size (d/cm)**						-1.265	0.206
	<4cm	55	14 (25.45)	21 (38.18)	20 (36.36)		
	≥4cm	19	3 (15.79)	6 (31.58)	10 (52.63)		
**Tumor recurrence**						-2.578	0.010*
	Yes	27	3 (11.11)	8 (29.63)	16 (59.26)		
	No	47	14 (29.79)	19 (40.43)	14 (29.79)		

@ 1: strong Chemerin expression + high TANs density group (%).

@ 2: strong Chemerin expression + low TANs density group/weak Chemerin expression + high TANs density group (%).

@ 3: weak Chemerin expression + low TANs density group (%).

P value was estimated by the Mann–Whitney test.

*P < 0.05 was considered to be statistically significant.

### Tissue Microarray and Double Staining Immunohistochemistry

The tissue microarray (TMA) used in this work was constructed as follows: First, representative areas located away from necrotic and hemorrhagic materials were premarked in paraffin-embedded wax blocks *via* H&E staining. Triplicates of 1-mm diameter cylinders from the centers of the tumors of 74 cases and the peritumoral noncancerous squamous epithelial tissues of 17 cases (designated as tumor and peritumoral tissues, respectively) were included in the TMA. Thus, several different TMA blocks were constructed. The blocks then were sectioned to a thickness of 4 µm and placed on slides that were coated with 3-aminopropyltriethoxysilane for immunohistochemistry (IHC).

A polymer double-staining kit (Zhongshan Golden Bridge Biotech, China) was used for staining. After the deparaffinization and gradient ethanol hydration of the sections, endogenous enzymes were inactivated by using 3% H_2_O_2_ for 30 min. The sections were heated in a water bath for heat-induced epitope retrieval in EDTA buffer (95°C) and cooled naturally at room temperature. After washing with PBS, the sections were incubated with anti-Chemerin (dilution of 1:100, Proteintech, China) or anti-CD15 (dilution of 1:150) antibodies overnight at 4°C in a humidity chamber. The polymer horseradish peroxidase detection system (ZSGB, China) in this work used DAB and GBI for visualization and hematoxylin for nuclear counter staining. The results showed that DAB-stained CD15+ was brown, GBI-stained Chemerin was red, and hematoxylin-stained cell nuclei were blue.

### Immunostaining

The evaluation criteria for the IHC results were as previously reported (6). Chemerin staining was evaluated on the basis of a semiquantitative scoring system. The intensity score represented the average intensity of the positive tumor cells (0, none; 1, weak; 2, intermediate; and 3, strong) (20). The proportion score represented the estimated proportion of positive tumor cells: 0: (<5%), 1: (5%–25%), 2: (26%–50%), 3: (51%–75%), and 4: (>75%). The proportion and intensity scores were then added to obtain a total score, which ranged from 0 to 7. All specimens were divided into two groups: weak expression, 0–3 points and strong expression, 4–7 points. For the CD15+ neutrophil count, positive cells in three cylinders with a diameter of 1 mm per patient were calculated and presented as the mean value of the triplicates (cells/core). The average value of CD15+ neutrophils in TMA was acquired, and the median of CD15+ neutrophils of the 74 samples was obtained as the cut-off value in subsequent analysis.

If the mean number of the triplicates was more than the median, the specimen was allocated into the high-density group and into the low-density group otherwise.

The specimens were divided into three groups to evaluate the expression of Chemerin and CD15+TANs density: the strong Chemerin expression+ high TANs density group (@1), the strong Chemerin expression+ low TANs density group/weak Chemerin expression+ high TANs density group (@2), and the weak Chemerin expression+ low TANs density group (@3).

### Cell Culture

OSCC lines (Cal27, SCC9, and SCC15) were purchased from Shanghai Institutes for the Chinese Academy of Sciences and cultured at 37°C in a humidified atmosphere of 5% CO_2_ in Dulbecco’s modified Eagle’s medium (DMEM) containing 10% FBS with 1% penicillin–streptomycin. For co-cultivation studies, 1 × 10^6^ OSCC cells and neutrophils (1:3 ratio) seeded 24 h before co-cultivation were added to the upper chamber of a Transwell chamber with a 0.4-μm porous polycarbonate membrane (Corning, Union City, CA, USA) or to the lower chamber. The OSCC cells were collected after 24–48 h.

### Extraction of Neutrophils From Peripheral Blood

Neutrophils were isolated from peripheral blood of healthy donors at the Affiliated Hospital of Qingdao University after written informed consent was obtained. In a 15 ml centrifuge tube, 5.0 ml of anticoagulated whole blood was layered on 5.0 ml of PolymorphPrep (Axis-Shield PoC AS, Norway).After centrifugation at 450 × *g* for 30 min, the mononuclear cell phase was discarded to isolate neutrophils. After centrifugation, two bands of white blood cells were obtained. The top band consisted of mononuclear cells, and the lower band consisted of polymorphonuclear neutrophils (PMNs). All the cell bands were collected, and the remaining RBCs were lysed with a hypotonic lysis program to obtain the pure PMNs population.

### Transwell Chemotaxis Assay

The cell chemotaxis assay was performed in a 24-well Transwell chamber with a polycarbonate membrane with a 3 mm pore size (Corning, USA). A total of 2×10^5^ neutrophils were suspended in 200 µl of serum-free medium in each upper compartment. Then, 600 µl of RPMI-1640 with 10% serum containing recombinant Chemerin (20, 50, 100, or 200 ng/ml) or 2×10^5^ OSCC cells (Cal27, SCC9, and SCC15) was added to the lower compartment. RPMI-1640 with 10% serum was added to the lower compartment of the control group. After incubation at 37°C for 24 h, the suspended neutrophils in the lower compartment were counted by using a cell counting plate. The chemotactic index for each group was calculated as follows: neutrophils count of each experimental group/neutrophils count of the control group × 100%.

### Enzyme-Linked Immunosorbent Assay

The concentration of Chemerin in the medium was measured *via* an enzyme-linked immunosorbent assay (ELISA, R&D Systems, USA) in accordance with the manufacturer’s instructions.

### Lentiviral Transduction

Lentiviruses were synthesized by GenePharma (Suzhou, China). The sequences of Chemerin-shRNA and scramble controls were as follows: Chemerin-shRNA1#, 5′-GCCCTTCCCAGCTGGAATATT-3′; Chemerin-shRNA2#, 5′-GCTTCTACTTCCCTGGACAGT-3′; negative control (Scr-shRNA), 5′-ACGUGACACGUUCGGAGAADTDT-3′. Chemerin was over-expressed by using an over-expression vector plasmid (Chemerin). Empty plasmids were used as the negative control (NC). Plasmids were purchased from GenePharma (GenePharma, China).

### Cell Viability Assay

For the cell co-culture, 3 × 10^3^ OSCC cells were seeded into each well of 96-well plates in the presence or absence of neutrophils (1:3 ratio). After 24 h, the neutrophils were removed from the co-culture system. Subsequently, methylthiazolyltetrazole (MTT) measurements were performed at different time points (0, 24, 48, 72, and 96 h). A total of 20 µl of MTT (5 mg/ml) was added into each well, and the cells were further incubated for 4 h. Then, the media were discarded, and 150 µl of DMSO was added. The plate was shaken at room temperature for 15 min, and absorbance was read at 490 nm on an automatic microplate reader (Bio-Tek Instruments, Winooski, VT, USA).

### Colony Formation Assay

After 24–48 h of co-culture or non-co-culture with neutrophils, the OSCC cells were cultured in a six-well plate at the density of 3000 cells/well for 2 weeks. The colonies that formed were fixed with methanol for 30 min and sequentially stained with 0.5% crystal violet for half an hour.

### Cell Cycle Analysis

After 24–48 h of co-culture or non-co-culture with neutrophils, the OSCC cells were washed with ice-cold PBS and harvested *via* trypsinization. After centrifugation for 5 min, the cells were washed with ice-cold PBS and fixed with 70% ethanol overnight at 4°C. RNaseA (20 μg/ml) was added to the cells for 30 min (at 37°C). Propidium iodine (50 µg/ml) was added to the cells in the dark. The cells were analyzed by using flow cytometry (BD Accuri C6).

### Cell Migration and Invasion Assay

The cell migration/invasion assay was performed in a 24-well Transwell chamber with a polycarbonate membrane with an 8 mm pore size (Corning, Union City, CA, USA). A total of 50 μl of diluted Matrigel (Matrigel: serum-free medium=1:8) was added into the upper chamber of the Transwell plates for the invasion assay, whereas the plates without Matrigel in their upper chambers were used for the migration assay. The treated cells were incubated in serum-free medium for 24 h. A total of 1.25–2.5 × 10^5^ neutrophils in 500 μl of RPMI-1640 containing 15% FBS were added to the lower chamber, and 5 × 10^4^ OSCC cells in 500 μl of DMEM containing 15% FBS were added to the upper chamber. After 24–48 h of incubation at 37°C, the cells that had migrated to the medium containing 15% serum in the lower compartment were stained with 0.5% crystal violet. The number of cells in five random microscope fields (100×) was counted.

### Quantitative Real-Time PCR

Total RNA extraction and quantitative real-time PCR (qRT-PCR)were performed as previously described (6). Primers were synthesized by the Shanghai Sangon Biological Engineering Technology & Services Co.

The primers used in this assay were:

Chemerin: 5′-AGACAAGCTGCCGGAAGAGG-3′(upper)

and 5′-TGGAGAAGGCGAACTGTCCA-3′(lower);

GAPDH: 5′-CGGAGTCAACGGATTTGGTCGTAT-3′(upper)

and 5′-AGCCTTCTCCATGGTGGTGAAGAC-3′(lower).

### Western Blot Analysis

Protein preparation and Western blot analysis were performed as described previously (33). The antibodies used in the study were as follows: Chemerin (Abcam, dilution of 1:500), GAPDH, E-cadherin, N-cadherin, Vimentin, p-JAK2, JAK2, Phospho-Rb (Abcam, dilution of 1:1000), p-STAT3, STAT3, Phospho-Rb (Abcam, dilution of 1:2000), E2F1, Cyclin D1, Cyclin E1 (Abcam, dilution of 1:1000), Slug and Snail (ProteinTech, dilution of 1:1000), and β-actin (ProteinTech, dilution of 1:4000).

### Statistical Analysis

All statistical analyses were performed by using SPSS 23.0 software. All values were presented as mean ± SD, and each experiment was performed at least three times. Mann–Whitney test was used for non-normally distributed data. Student’s *t* test was used for data that were normally distributed. Differences were considered statistically significant at *P*< 0.05, *P*< 0.01, and *P*< 0.001.

## Results

### Immunohistochemical Analysis of TANs Infiltration and Chemerin Expression and Their Correlation With Clinicopathological Parameters

**Figure 1:**
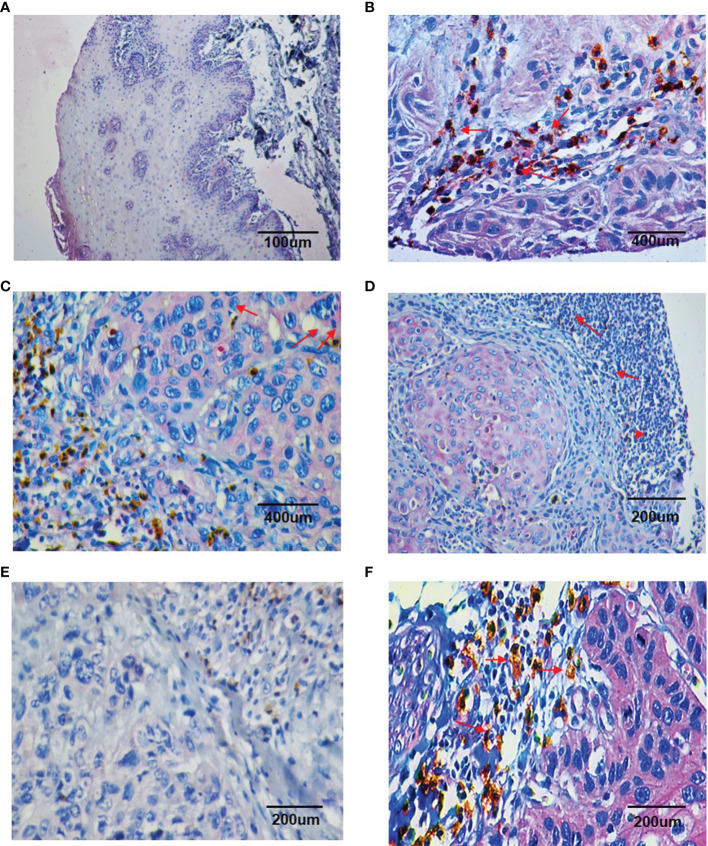
Double staining immunohistochemistry results of CD15 + TANs and Chemerin. **(A–D)** The results showed more CD15 + TANs (red arrow) infiltrated in OSCC tissues **(B–D)** than in peritumoral tissues **(A)**, with TANs predominantly observed in stroma around the carcinoma nests **(B)**, within the carcinoma nests **(C)** and in the borderline of tumor invasion **(D)**. (A, 100×; B, C, 400×; D, 200×). Chemerin (pink color) was absent or weakly expressed in peritumoral non-neoplastic tissues **(A)**, whereas its expression was upregulated in cancer tissues compared with peritumoral tissues and located mainly in the cytoplasm of tumor cells **(B–D)**. **(E, F)** The relationship of Chemerin expression on tumor cells and infiltration of TANs. In negative or weak Chemerin expression tumors, there were relatively fewer TANs **(E)**; while in strong Chemerin expression tumors, there were more TANs (red arrow) **(F)**. (E, F, 200×). Brown: CD15 + (DAB staining), Pink: Chemerin (GBI staining).

Double IHC results revealed that Chemerin and CD15+ TANs co-localized in clinical OSCC specimens. The IHC results demonstrated that CD15+ TANs infiltration in OSCC tissues (**Figures 1B** **–D**) was greater than that in peritumoral tissues (**Figure 1A**). TANs were predominantly observed in the stroma around carcinoma nests (**Figure 1B**), within carcinoma nests (**Figure 1C**), and in the borderline of tumor invasion (**Figure 1D**). Tumors with negative or weak Chemerin expression had low TANs infiltration (**Figure 1E**), whereas those with strong Chemerin expression had high TANs infiltration (red arrow) (**Figure 1F**). The number of CD15+ TANs outside the blood vessels ranged from 1 to 1053.5 in each 1mm-diameter tissue sample. The median density was 59.5/core. If the mean number of the triplicates exceeded 59.5, the sample was allocated into the high-density group. Otherwise, it was allocated into the low-density group. In addition, the IHC results showed that in OSCC, Chemerin expression was heterogeneous. Among the 74 cases of OSCC, 25 (33.78%) had weak Chemerin expression and 49 (66.22%) had strong Chemerin expression.

In tumor tissues, strong Chemerin expression+ high TANs density was associated with high clinical stage (*P* < 0.001), lymph node metastasis (*P* < 0.001), and tumor recurrence (*P* = 0.01) (**Table 1**). As shown in **Table 2**, Spearman’s rho coefficient revealed that Chemerin expression was positively correlated with CD15+ TANs infiltration in OSCC (*P* = 0.017).

**Table 2:** The relationship between Chemerin expression and TANs density in OSCC tissues.

Chemerin expression	CD15 + TANs density		Spearman’s rho Coefficient test	
	**Low**	**High**	**r**	***P* value**
**Low**	**16**	**9**	**0.309**	0.017*
**High**	**20**	**29**		

*P < 0.05 was considered to be statistically significant.

### Analysis of the Influence of TANs Infiltration and Chemerin Expression on the Survival of Patients With OSCC

We followed up all 74 patients to evaluate the effect of TAN infiltration and Chemerin expression on the survival of patients with OSCC. The median follow-up time was 96 months. Within the observation period, 37 patients died from cancer.

**Figure 2:**
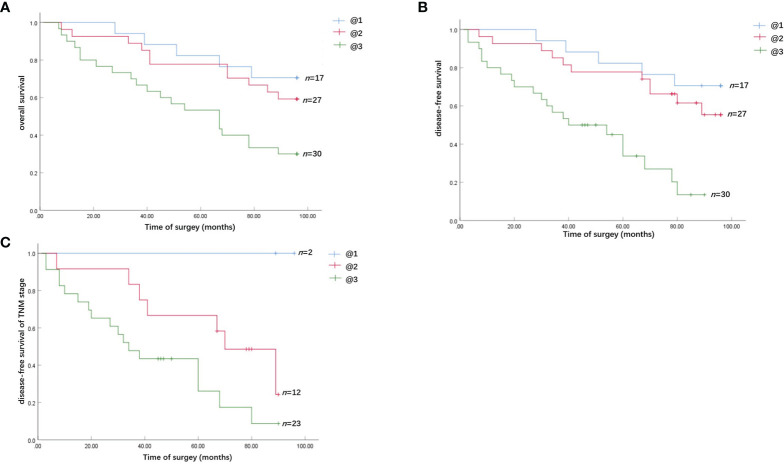
Survival analysis of the effect of Chemerin expression and TANs infiltration on OSCC patients. **(A)** The overall cancer-related survival of OSCC patients in the strong Chemerin expression + high TANs density group was significantly reduced, compared with the weak Chemerin expression + low TANs density group (*P* = 0.008, log-rank test) and strong Chemerin expression + low TANs density group/weak Chemerin expression + high TANs density group (*P* = 0.017, log-rank test). **(B)** The OSCC patients in the strong Chemerin expression + high TANs density group also had significantly reduced disease-free survival related to cancer, compared with the weak Chemerin expression + low TANs density group (*P* = 0.000, log-rank test) and strong Chemerin expression + low TANs density group/weak Chemerin expression + high TANs density group (*P* = 0.001, log-rank test). **(C)** In TNM stage III and IV OSCC patients, the cancer-related disease-free survival of OSCC patients in the strong Chemerin expression + high TANs density group was significantly reduced (*P* = 0.045, log-rank test).

Kaplan–Meier survival analysis revealed that strong Chemerin expression+ high neutrophil density (**Figures 2A, B****)** and advanced clinical stage (**Figure 2C**) were associated with the short cancer-related survival of patients with OSCC. Tumor size (*P* = 0.591), tumor differentiation (*P* = 0.312), tumor recurrence (*P* = 0.123), patient gender (*P* = 0.597), and age (*P* = 0.592) had no effect on cancer-related survival. We performed multivariate survival analysis by using a Cox proportional hazards model to analyze whether the above parameters are independent prognostic factors for the survival of patients with OSCC. The results showed that strong Chemerin expression and high CD15+ TANs density were independent prognostic factors for patients with OSCC (**Tables 3** and **4**).

**Table 3:** Univariate and multivariate overall survival analysis in OSCC patients.

Variables	Regression coefficient	Univariate analysis		Regression coefficient	Multivariate analysis	
** **		Hazard ratio (95% CI)	*P *value		Hazard ratio (95% CI)	*P* value
**Sex**	-0.174	0.841 (0.415~1.702)	0.630			
**Age**	0.313	1.367 (0.716~2.612)	0.343			
**TNM stage**	0.934	2.544 (1.292~5.009)	0.007*	1.246	3.477 (0.76~15.894)	0.108
**Differentiation**	-0.218	0.804 (0.422~1.534)	0.508			
**Lymph node metastasis**	0.841	2.319 (1.2~4.482)	0.012*	-0.718	0.488 (0.101~2.349)	0.371
**Tumor size**	0.148	1.16 (0.561~2.396)	0.689			
**Tumor recurrence**	0.369	1.446 (0.753~2.775)	0.268			
**The expression of Chemerin, CD15 + TANs density**	0.691	1.995 (1.246~3.195)	0.004*	0.598	1.810 (1.056~3.133)	0.031*

CI,  Confidence interval.

*P < 0.05 was considered to be statistically significant.

**Table 4:** Univariate and multivariate disease-free survival analysis in OSCC patients.

Variables	Regression coefficient	Univariate analysis		Regression coefficient	Multivariate analysis	
** **		Hazard ratio (95% CI)	*P* value		Hazard ratio (95% CI)	*P* value
**Sex**	-0.191	0.826 (0.408~1.674)	0.597			
**Age**	0.177	1.194 (0.624~2.285)	0.592			
**TNM stage**	1.907	2.996 (1.508~5.951)	0.002*	1.351	3.861 (0.838~17.79)	0.083
**Differentiation**	-0.335	0.716 (0.374~1.368)	0.312			
**Lymph node metastasis**	1.022	2.779 (1.423~5.427)	0.003*	-0.773	0.462 (0.095~2.236)	0.337
**Tumor size**	0.199	1.221 (0.591~2.523)	0.591			
**Tumor recurrence**	0.516	1.675 (0.87~3.225)	0.123			
**The expression of Chemerin, CD15 + TANs density**	0.968	2.634 (1.588~4.367)	<0.001*	0.877	2.405 (1.353~4.273)	0.003*

CI, Confidence interval.

*P < 0.05 was considered to be statistically significant.

### OSCC Cells Expressing Chemerin Attract Neutrophils *In Vitro*

**Figure 3:**
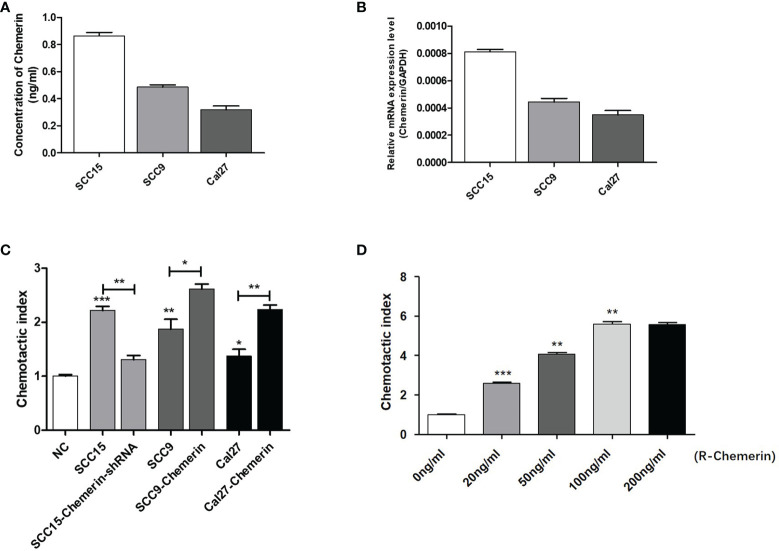
OSCC cells expressing Chemerin attract neutrophils *in vitro*. **(A)** Supernatant Chemerin levels in OSCC cell lines were detected by ELISAs. **(B)** mRNA expression levels of Chemerin in OSCC cell lines were detected by qRT-PCR. **(C)** The effect of OSCC cells on neutrophils’ chemotaxis was assayed using a transwell system. **(D)** The effect of R- Chemerin on neutrophils’ chemotaxis was assayed using a transwell system at various concentrations (0-200 ng/ml). *P < 0.05; **P < 0.01; ***P < 0.001.

On the basis of the IHC results, we explored whether Chemerin can attract neutrophils *in vitro via* Transwell assay. The results of ELISAs (**Figure 3A**) and qRT-PCR (**Figure 3B**) showed that all three cell lines had a certain level of Chemerin expression and secretion. As depicted in **Figure 3C**, compared with the blank group, all three OSCC cells demonstrated improved chemotaxis to neutrophils (*P*< 0.05). Among the three cell lines, SCC15 demonstrated the most effective chemotaxis and the highest level of Chemerin (**Figures 3A** **–C**).The chemotactic effect of the SCC15-Chemerin-shRNA group of cells on neutrophils was weaker than that of the SCC15 group. By contrast, the chemotactic effect of the SCC9-Chemerin and Cal27-Chemerin groups was stronger than that of the SCC9 and Cal27 groups. After Chemerin knockdown, the chemotactic effect of SCC15 on neutrophils weakened, and after Chemerin over-expression, that of SCC9 and Cal27 on neutrophils strengthened (**Figure 3C**). R-Chemerin was also used to verify the chemotactic effect of Chemerin on neutrophils. The results showed that R-Chemerin improved the chemotaxis of neutrophils in a concentration-dependent manner (**Figure 3D**).

### Lentiviral Transfection

**Figure 4:**
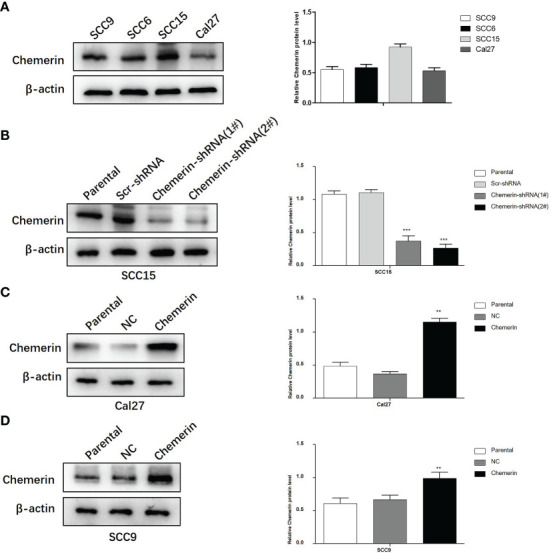
The silencing and over-expression efficiency of Chemerin in OSCC cell lines. **(A)** Western blot analysis of Chemerin protein expression in SCC9, SCC6, SCC15 and Cal27 cells. β-actin was used as the control. **(B)** The knockdown efficiency of Chemerin-shRNA (1# and 2#) in SCC15 cell. In the following experiments, Chemerin-shRNA 2# was chosen because of its higher efficiency in silencing Chemerin. Scr-shRNA was used as a negative control. **(C, D)** SCC9 and Cal27 cells were used to over-express Chemerin by lentivirus, and Western blot analysis showed the expression of Chemerin increased after Chemerin transfection (*P < 0.05; **P < 0.01; ***P < 0.001). NC was used as a negative control. All data were presented as mean ± SD. All the experiments were repeated three times.

Chemerin expression in the SCC9, SCC6, SCC15, and Cal27 cell lines was examined by using Western blot analyses (**Figure 4A**). SCC15 cells were selected for the knock-out of Chemerin with Chemerin-shRNA1# and 2# given their high Chemerin expression levels. The results showed that the Chemerin-shRNA2# was more effective than Chemerin-shRNA1# (**Figure 4B**). Therefore, Chemerin-shRNA 2# was chosen for the follow-up experiment. Meanwhile, given their low Chemerin expression levels, the Cal27 and SCC9 cell lines were transfected with the Chemerin over-expression lentivirus (**Figures 4C, D****)**.

### Neutrophils Promote OSCC Cell Proliferation Through Chemerin

**Figure 5:**
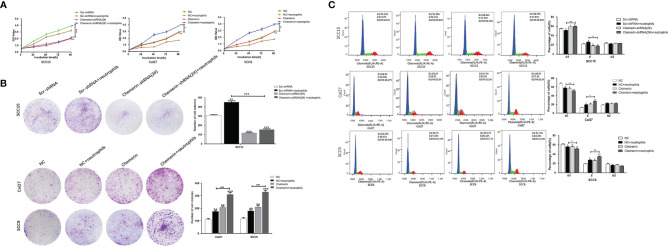
Neutrophils promote cell proliferation through Chemerin. **(A)** Cell growth was analyzed using the MTT assay. Absorbance of 490 nm was measured. The data were presented as the means of six separated experiments, each performed in triplicate. **(B)** Colony formation in each group was photographed and colony numbers were illustrated in histogram. **(C)** Flow cytometry revealed the distribution of cell phases in the OSCC cell lines. Data were shown as mean ± SD from three independent experiments (*P < 0.05; **P < 0.01; ***P < 0.001).

MTT and colony formation assays were performed to explore the effect of Chemerin and neutrophils on cell proliferation. OSCC cells were transfected with Chemerin-targeting knock-down (over-expression) and/or subjected to combined treatment with neutrophils. The results showed that the growth rate of the neutrophils group was significantly higher than that of the Scr-shRNA group (NC group). The growth rate of the Cal27 and SCC9 cells in the Chemerin over-expression group was significantly higher than that of the Cal27 and SCC9 cells in the NC group, and the growth rate of the Cal27 and SCC9 cells in the Chemerin+ neutrophils group was higher than that of the Cal27 and SCC9 cells in the neutrophils group (**Figure 5A**). Furthermore, the growth rate of the SCC15 cells in the Chemerin-shRNA (2#) group was significantly lower than that of the SCC15 cells in the Scr-shRNA group. The effect of neutrophils on cell proliferation was abolished after Chemerin knockdown (**Figure 5A**). The colony numbers of the Scr-shRNA, Scr-shRNA+ neutrophils, Chemerin-shRNA (2#), and Chemerin-shRNA (2#)+ neutrophils groups of the SCC15 cells were 304.29 ± 35.42, 459.66 ± 38.73,119.76± ± 32.91, and 154.15 ± 34.35, respectively (**Figure 5B**). The colony numbers of the Cal27 cells in the NC, NC+ neutrophils, Chemerin, and Chemerin+ neutrophils groups were 110.32 ± 31.41, 176.62 ± 35.53, 200.72 ± 38.91, and 301.35 ± 44.75, respectively, and those of the SCC9 cells in the Scr-shRNA, Scr-shRNA+ neutrophils, Chemerin-shRNA (2#), and Chemerin-shRNA (2#)+ neutrophils groups were 121.62 ± 35.39, 182.42 ± 31.59, 202.41 ± 42.21, and 331.18 ± 38.75, respectively (**Figure 5B**). These findings indicated that neutrophils promoted the proliferation of OSCC cells *via* Chemerin.

Flow cytometry was performed to further study the effects of Chemerin and neutrophils on the cell cycle. The cell phase distributions of the Scr-shRNA, Scr-shRNA+neutrophils, Chemerin-shRNA (2#), and Chemerin-shRNA (2#)+ neutrophils groups of SCC15 cells were as follows (**Figure 5C**): G1 phase: 55.13% ± 1.16%, 51.58% ± 2.71%, 58.34 ± 1.32% and 59.55% ± 2.15%; S phase: 21.67% ± 1.45%,26.11 ± 2.81%, 17.52% ± 1.74% and 17.81% ± 1.36%; and G2/M phase: 23.2% ± 2.19%, 22.31% ± 3.41%, 24.14% ± 2.08%, and 22.64% ± 1.95%. The proportion of the G1 phase was significantly decreased and the G2/M phase ratio was significantly increased in the Scr-shRNA group compared with those in the neutrophils group. Compared with the Scr-shRNA group, the Chemerin-shRNA (2#) group had a significant reduction in the proportion of cells in the G1 phase and a significant increase in the proportion of cells in the G2/M phase, indicating significant G2/M arrest. The effect of neutrophils on the cell cycle was abolished after Chemerin knock-down. The cell phase distributions of NC, NC+ neutrophils, Chemerin, and Chemerin+ neutrophils Cal27 cells were determined to further confirm these findings and were as follows (**Figure 4C**): G1 phase: 68.8% ± 2.96%, 58.2% ± 2.01%, 57.5% ± 2.30%, and 49.5% ± 2.110%; S phase: 12.93% ± 2.29%,19.5% ± 1.87%, 20.3% ± 2.74%, and 27.3% ± 1.21%; and G2/M phase: 18.27% ± 2.01%, 22.3% ± 1.65%, 22.2% ± 2.35%, and 23.2% ± 2.25%. The cell phase distributions of the NC, NC+ neutrophils, Chemerin, and Chemerin+ neutrophils SCC9 cells were as follows (**Figure 5C**): G1 phase: 62.20% ± 1.26%, 56.70% ± 1.01%, 56.1% ± 1.30%, and 51.8% ± 1.45%; S phase: 18.40% ± 1.59%,27.10% ± 1.87%, 26.2% ± 1.74%, and 34.2% ± 2.71%; and G2/M phase: 19.1% ± 2.09%, 16.2% ± 3.05%, 17.7% ± 2.38%, and 14.0% ± 2.35%.

### Neutrophils Promote the Migration, Invasion, and EMT of OSCC Cells Through Chemerin

**Figure 6:**
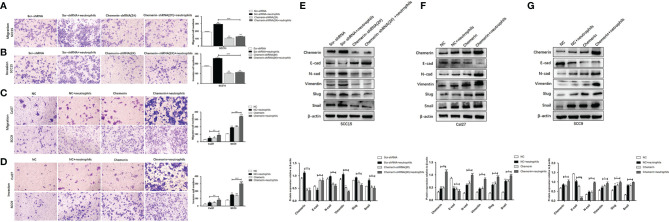
Neutrophils promote migration, invasion and EMT of OSCC cells through Chemerin. **(A, B)** Transwell and invasion assays showed migration and invasion of SCC15 cells in the Scr-shRNA, Scr-shRNA + neutrophils, Chemerin-shRNA (2#), and Chemerin-shRNA (2#) +neutrophils groups. **(C, D)** Migration and invasion assay of Cal27 and SCC9 cells in NC, NC + neutrophils, Chemerin, and Chemerin + neutrophils groups. **(E–G)** Western blot of Chemerin, E-cadherin, N-cadherin, Vimentin, Slug and Snail. All data were presented as mean ± SD values from three independent experiments, each performed in triplicates (*P < 0.05; **P < 0.01; ***P < 0.001).

Transwell assay was used to further investigate the migration and invasion of OSCC cells. The results showed that in the Scr-shRNA, Scr-shRNA+ neutrophils, Chemerin-shRNA (2#), and Chemerin-shRNA (2#)+ neutrophils groups, the numbers of migrated SCC15 cells were 188.67 ± 9.07, 295.67 ± 13.23, 107.67 ± 5.86, and 127.67 ± 10.02 (**Figure 6A**), respectively, and the numbers of invasive SCC15 cells were 173.56 ± 6.11, 255.00 ± 5.00, 103.47 ± 7.37 and 113.33 ± 6.65 (**Figure 6B**), respectively. These data suggested that neutrophils promoted cell migration and invasion and that the down-regulation of Chemerin significantly inhibited the migration and invasion of SCC15 cells. Furthermore, the effect of neutrophils on cell migration and invasion was abolished after Chemerin knockdown.

Transwell assays were performed on Chemerin-over-expressing Cal27 and SCC9 cells to further confirm these results. In the NC, NC+ neutrophils, Chemerin, and Chemerin+ neutrophils groups, the numbers of migrated Cal27(SCC9) cells were 29.33 ± 4.14, 48.67 ± 7.12, 49.00 ± 5.62, and 88.67 ± 5.60 (105.32 ± 9.76, 189.67 ± 10.06, 200.32 ± 9.56, and 339.14 ± 7.02) (**Figure 6C**), respectively, and the numbers of invasive Cal27(SCC9) cells were 25.34 ± 6.13, 45.34 ± 5.51, 44.67 ± 6.66, and 81.52 ± 8.08(79.00 ± 6.56, 151.01 ± 9.07, 143.07 ± 7.94, and 301.67 ± 11.50), respectively (**Figure 6D**). Therefore, neutrophils may promote the migration and invasion of OSCC cells *via* Chemerin.

EMT, a well-characterized embryological process, has been identified to play a critical role in tumor metastasis (34–36). We cultured OSCC cells with neutrophils in a previously described co-culture system to explore the role of neutrophils and Chemerin in mediating EMT in OSCC cells.

Chemerin, Slug, Snail, E-cadherin, N-cadherin, and Vimentin were detected in OSCC *via* Western blot analysis to further examine the effect of Chemerin and neutrophils on EMT. Neutrophils decreased the expression of E-cadherin and increased the expression levels of Chemerin, Slug, Snail, N-cadherin, and Vimentin in the three OSCC cell lines relative to in the control group. Compared with those in the Scr-shRNA group, the expression of E-cadherin increased and the expression levels of Chemerin, N-cadherin, Vimentin, Slug, and Snail decreased in the Chemerin-shRNA (2#) SCC15 cells. Moreover, the effects of neutrophils on Chemerin, E-cadherin, N-cadherin, Vimentin, Slug, and Snail significantly decreased after Chemerin knock-down (**Figure 6E**). This correlation was then confirmed in Cal27 and SCC9 cells (**Figures 6F, G****)**, in which Chemerin over-expression further promoted the expression of EMT-related proteins in the neutrophils group. Therefore, neutrophils promoted the EMT of OSCC cells and further promoted the migration and invasion of OSCC cells. This effect may be mediated by Chemerin.

### Neutrophils Activate the JAK2/STAT3 Signaling Pathway in OSCC Cells Through Chemerin

**Figure 7:**
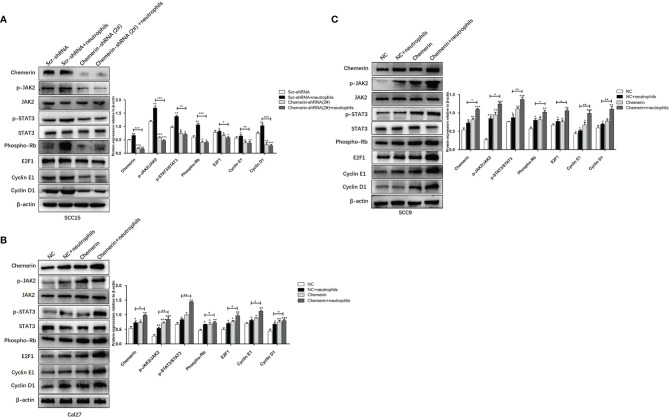
Neutrophils activate the JAK2/STAT3 signaling pathway in OSCC cells through Chemerin. **(A)** The levels of JAK2, p-JAK2, STAT3, p-STAT3, Phospho-Rb, E2F1, CyclinE1, CyclinD1 protein of SCC15 in the Scr-shRNA, Scr-shRNA + neutrophils, Chemerin-shRNA (2#), and Chemerin-shRNA (2#) +neutrophils groups. **(B, C)** The levels of JAK2, p-JAK2, STAT3, p-STAT3, Phospho-Rb, E2F1, CyclinE1, CyclinD1 protein of Cal27 and SCC15 in NC, NC + neutrophils, Chemerin, and Chemerin + neutrophils groups. All data were presented as mean ± SD. All the experiments were repeated three times, each performed in triplicates (*P < 0.05; **P < 0.01; ***P < 0.001).

We used using Western blot analysis to examine the expression levels of JAK2, p-JAK2, STAT3, p-STAT3, Phospho-Rb, E2F1, CyclinE1, and CyclinD1 in the Scr-shRNA, Scr-shRNA+ neutrophils, Chemerin-shRNA (2#), and Chemerin-shRNA (2#)+neutrophils groups of SCC15 cells and the NC, NC+ neutrophils, Chemerin, and Chemerin+ neutrophils groups of Cal27(SCC9) cells to investigate whether JAK2/STAT3 signaling is involved in the interaction between neutrophils and Chemerin in OSCC cells in the co-culture system. We found that neutrophils increased the expression levels of p-JAK2, p-STAT3, Phospho-Rb, E2F1, CyclinE1, and CyclinD1 in the three OSCC cells relative to those in the control group. p-JAK2, p-STAT3, Phospho-Rb, E2F1, CyclinE1, and CyclinD1 levels decreased in the Chemerin-shRNA (2#) SCC15 cells relative to those in the Scr-shRNA group. Moreover, the effects of neutrophils on p-JAK2, p-STAT3, Phospho-Rb, E2F1, CyclinE1, and CyclinD1 significantly decreased after Chemerin knockdown (**Figure 7A**). This correlation was then confirmed through Chemerin over-expression in Cal27 and SCC9 cells, in which Chemerin over-expression further promoted the expression of proteins in the neutrophils group (**Figures 7B, C****)**. All these data indicated that neutrophils may regulate the JAK2/STAT3 signaling pathway through Chemerin to promote the proliferation and invasion of OSCC cells.

## Discussion

Neutrophils are the most abundant subpopulation of leukocytes that provide the first line of defense against invading pathogens. Studies have found that TANs have multiple functions in the tumor microenvironment (6). Depending on the tumor microenvironment, TANs may affect tumor progression through cytokines and chemokines with tumor-promoting or antitumor functions (7). Chemerin is a chemoattractant and a novel multifunctional adipokine. It plays an important role in regulating inflammation, angiogenesis, fat metabolism, cell proliferation, migration, and chemotaxis (37–40). However, the possible roles of Chemerin expression and neutrophils in OSCC have not been experimentally verified.

Chemokines are important components of cancer-related inflammatory conditions that promote tumor progression in many ways; these conditions include leukocyte recruitment and function, tumor cell proliferation, invasion, and metastasis (41–43). Previous research has demonstrated that Chemerin induces the chemotaxis of immune cells, such as immature dendritic cells, resident macrophages, and cytotoxic natural killer cells, to inflammatory sites (44). We used the Transwell assay to explore the possible chemotactic effects of Chemerin from tumor cells on neutrophils. Our results showed that all three OSCC cell lines exhibited enhanced chemotaxis to neutrophils, and SCC15, which had the highest Chemerin level, had the greatest chemotactic effect. R-Chemerin also improved chemotaxis to neutrophils in a concentration-dependent manner. Additionally, our double-staining IHC results revealed that Chemerin expression demonstrated great heterogeneity in OSCC tissues and that TANs infiltration was increased in areas with strong Chemerin expression. Spearman’s rho coefficient test indicated that Chemerin expression was positively related to the density of CD15+ TANs in OSCC tissues. Strong Chemerin expression+ high TANs density was associated with lymph node metastasis, high clinical stage, and tumor recurrence. This association indicated that strong Chemerin expression+ high TANs density may predict poor clinical outcomes. Moreover, survival analysis illustrated that strong Chemerin expression+ high TANs density was related to shortened overall and disease-free survival and that each factor was an independent prognostic factor of patients with OSCC. These results implied that Chemerin over-expression in OSCC may attract additional neutrophils to tumor sites and promote neutrophil-mediated tumor progression.

EMT is a key step for tumor cells to obtain enhanced invasive and metastatic capabilities; this step is characterized by the loss of connections and the acquisition of mesenchymal properties, such as motility and invasiveness, by epithelial cells (45). Tumor cells use EMT as an intermediate phenotype to achieve self-renewal and adapt to their microenvironment (46, 47). Previous works have found that EMT can be induced not only by the loss of cell contact (for example, due to the degradation of the basement membrane or other changes in the microenvironment) but also by a variety of cytokines, such as TGF-β (48). Neutrophils can induce EMT in OSCC. However, the molecular mechanism of this effect is poorly understood. In the tumor microenvironment, a large number of cytokines and chemokines, such as IL-1β, IL-6, IL-8, and TNFα (49), can also induce EMT in OSCC. However, the current mechanism of Chemerin+ neutrophils polarization and its role in the progression of OSCC remains unclear. In the present study, we showed that neutrophils induced EMT in OSCC cells through Chemerin; this process was characterized by the decreased expression of E-cadherin and the increased expression of Slug, Snail, N-cadherin, and Vimentin. These EMT changes may help enhance the capability of OSCC cells to move actively. This enhancement is demonstrated by the increase in migratory and invasion capabilities triggered by neutrophils.

Chemerin is positively correlated with the activation of the STAT3 signaling pathway (50). The JAK signaling pathway promotes the activation of STAT3 and Tyr705 phosphorylation (27, 50, 51). A growing body of evidence indicates that the activation of the JAK2/STAT3 signaling pathway by chemokines or cytokines plays a positive role in tumor growth and progression. However, the role of the JAK2/STAT3 signaling pathway in OSCC has been poorly addressed. Our study demonstrated that the knockdown of Chemerin significantly impaired neutrophils-induced migration, invasion, and proliferation, as well as the neutrophils-induced EMT of OSCC cells *in vitro*. We also found that neutrophils activated JAK2/STAT3 signaling through Chemerin and then up-regulated JAK2/STAT3 signaling target genes, including Phospho-Rb, E2F1, CyclinE1, and CyclinD1 (52–55), which are highly expressed in OSCC. At the same time, neutrophils affected the cell cycle through Chemerin. In this study, we discovered that that knockdown of Chemerin only partially inhibited JAK2 or STAT3 phosphorylation, indicating that Chemerin partially contributed to the tumor-promoting effect of neutrophils on OSCC cells. Although the involvement of other growth factors and/or cytokines cannot be ruled out, in general, Chemerin is an important mediator of the tumor-promoting effect of neutrophils. In OSCC, Chemerin may promote EMT, tumorigenesis, and development by activating the JAK2/STAT3 signaling pathway.

Collectively, our results provided support for the first time that Chemerin over-expression and neutrophils abundance are associated with the poor clinical outcomes of patients with OSCC. Chemerin over-expression and neutrophils infiltration were the prognostic factors of poor clinical outcomes. In addition, on the one hand, neutrophils/chemerin activated JAK2/STAT3 pathway and upregulated its down stream cycle-related proteins to promote tumor proliferation. On the other hand, neutrophils/chemerin induced EMT of OSCC also through the JAK2/STAT3 pathway and further promoted tumor migration and invasion *via* EMT. So we believe that neutrophils may promote tumor progression (proliferation and invasion) *via* regulating EMT and JAK2/STAT3 signaling through Chemerin in Oral squamous cell carcinoma.

## Data Availability Statement

The raw data supporting the conclusions of this article will be made available by the authors, without undue reservation.

## Ethics Statement

This study was performed in line with the principles of the Declaration of Helsinki. Informed consent was obtained from all individual participants included in the study. Written informed consent for publication was obtained from the patients enrolled in the study. Approval was granted by the ethics committee of The Affiliated Hospital of Qingdao University (10.14.2020/QYFYWZLL25964). The patients/participants provided their written informed consent to participate in this study.

## Author Contributions

XH and NW conceived and designed the study. FX, YF, FG, SG and XZ collected clinical data. XH and NW wrote the manuscript. All authors have reviewed the final version of the manuscript and approved its submission for publishing.

## Funding

This study was funded by NW (National Natural Science Foundation of China, Grant Number: 81702677) and CW (National Natural Science Foundation of China, grant 81602320).

## Conflict of Interest

The authors declare that the research was conducted in the absence of any commercial or financial relationships that could be construed as a potential conflict of interest.

## Publisher’s Note

All claims expressed in this article are solely those of the authors and do not necessarily represent those of their affiliated organizations, or those of the publisher, the editors and the reviewers. Any product that may be evaluated in this article, or claim that may be made by its manufacturer, is not guaranteed or endorsed by the publisher.
